# Rapid maxillary expansion in mouth breathers: a short-term skeletal and soft-tissue effect on the nose^☆^

**DOI:** 10.1016/j.bjorl.2017.01.009

**Published:** 2017-02-24

**Authors:** Fauze Ramez Badreddine, Reginaldo R. Fujita, Fabio Eduardo Maiello Monteiro Alves, Mario Cappellette

**Affiliations:** Universidade Federal de São Paulo (UNIFESP), São Paulo, SP, Brazil

**Keywords:** Maxillary expansion, Palatal expansion technique, Nasal cavity, Nose, Expansão da maxila, Técnica de expansão palatal, Cavidade nasal, Nariz

## Abstract

**Introduction:**

Rapid maxillary expansion can change the form and function of the nose. The skeletal and soft tissue changes can influence the esthetics and the stability of the results obtained by the procedure.

**Objective:**

The aim of this retrospective study was to evaluate the short-term effects of rapid maxillary expansion on the skeletal and soft tissue structures of the nose, in mouth-breathing patients, using a reliable and reproducible, but simple methodology, with the aid of computed tomography.

**Methods:**

A total of 55 mouth-breathing patients with maxillary hypoplasia were assessed and were divided into an experimental group treated with rapid maxillary expansion(39 patients, 23 of which were male and 16 female, with an average age of 9.7 years and a standard deviation of 2.28, ranging from 6.5 to 14.7 years) and a control group (16 patients, 9 of which were male and 7 female, with an average age of 8.8 years, standard deviation of 2.17, ranging from 5.11 to 13.7 years). The patients of the experimental group were submitted to multislice computed tomography examinations at two different points in time: (T1) pre-rapid maxillary expansion and (T2) three months after the procedure. The control group underwent to the same exams at the same intervals of time. Four skeletal and soft tissue variables were assessed, comparing the results of T1 and T2.

**Results:**

There was in the experimental group a significant increases in all the skeletal and soft tissue variables (*p* < 0.05) but no significant alteration was found in the control group. When comparing the experimental group and the control group, the most important change occurred in the width of the pyriform aperture (*p* < 0.001).

**Conclusion:**

Rapid maxillary expansion is capable of altering the shape and function of the nose, promoting alterations in skeletal and soft tissue structures. This kind of study may, in the future, permit the proper planning of esthetic procedures at the tip and base of the nose and also the performance of objective measurements in early or late surgical outcomes.

## Resumo

**Introdução:**

A expansão rápida da maxila pode alterar a forma e a função do nariz. As alterações do esqueleto e dos tecidos moles podem influenciar a estética e a estabilidade dos resultados obtidos através deste procedimento.

**Objetivo:**

Avaliar, em curto prazo, os efeitos da expansão rápida da maxila sobre as estruturas esqueléticas e tegumentares do nariz em pacientes respiradores orais por meio de uma metodologia confiável e reprodutível, porém simples, com a ajuda da tomografia computadorizada.

**Método:**

Foram avaliados 55 pacientes respiradores orais com hipoplasia maxilar que foram divididos em grupo experimental tratado com expansão rápida da maxila (39, 23 do sexo masculino e 16 do feminino, com média de 9,7 anos e desvio padrão de 2,28, variação de 6,5 a 14,7 anos) e um grupo controle (16 pacientes, nove do sexo masculino e sete do feminino, com média de 8,8 anos, desvio padrão de 2,17, variação de 5,11-13,7 anos). Os pacientes do grupo experimental foram submetidos a exames de tomografia computadorizada multislice em dois tempos distintos: (T1) pré-expansão rápida da maxila e (T2) três meses após o procedimento. O grupo controle foi submetido aos mesmos exames nos mesmos intervalos de tempo. Foram avaliadas quatro variáveis esqueléticas e quatro tegumentares comparando-se os resultados de T1 e T2.

**Resultados:**

O grupo experimental apresentou aumentos significativos em todas as variáveis esqueléticas e de tecidos moles (*p* < 0,05), mas não houve alterações significativas no grupo controle. Ao comparar-se o grupo experimental e o grupo controle, foi observado que a alteração mais importante ocorreu na largura da abertura piriforme (*p* < 0,001).

**Conclusão:**

A expansão rápida da maxila é capaz de alterar a forma e a função do nariz, promovendo alterações nas estruturas esqueléticas e dos tecidos moles. Esse tipo de estudo pode, no futuro, permitir o planejamento adequado de procedimentos estéticos na ponta e base do nariz e também a realização de medidas objetivas em resultados cirúrgicos iniciais ou tardios.

## Introduction

It is well known that the nasal cavity plays an important role in respiratory physiology,^1^, ^2^ exerting a fundamental influence on facial growth and development and occlusion.^3^, ^4^ In view of the strong relationship that exists between the nasal cavity and the maxilla, and as the maxillary bone forms around 50% of the anatomic structure of the nasal cavity,^5^, ^6^ changes to the maxilla could also be the cause of nasal obstructions, the transverse deficiencies, which include maxillary hypoplasia, being the most frequently observed.^2^, ^7^

Therefore, the occurrence of maxillary hypoplasia together with respiratory problems, mainly nasal obstruction, has attracted the attention of various researchers to the possibility of these events harboring an intimate, mutual relationship.^8^, ^9^, ^10^ RME, in addition to rectifying occlusion, may have an influence on respiratory nasal activity,^1^, ^2^, ^7^, ^11^ as well as promoting positive changes in cervical posture and position of the head as a result of the smaller airway resistance.^12^, ^13^

From the earliest evidence of the effects of RME on the nasal cavity^8^, ^9^ various studies have been undertaken focusing on skeletal changes^5^, ^7^, ^11^, ^14^, ^15^, ^16^, ^17^, ^18^, ^19^, ^20^, ^21^, ^22^, ^23^, ^24^, ^25^, ^26^, ^27^, ^28^, ^29^, ^30^, ^31^, ^32^ the first specific study on the repercussion of RME on the soft tissue of the nasal cavity was only conducted as recently as 1999.^33^ Since this time, few studies have assessed the modifications to the facial soft tissue that have a tendency to stretch, accompanying the changes in the hard tissue due to bone expansion.^9^, ^23^, ^33^, ^34^, ^35^ The soft tissue, in addition to the esthetic impact it may have on the face,^36^ may also have a primary role in the maintenance and stability of occlusal and respiratory function after the RME. This happens because, after 3–4 months with a retainer, there could still be a lack of adaptation of the soft tissue, including that of the cheeks, which could become a recurrence factor.^9^, ^23^, ^33^ Moreover, there could also be losses in occlusal stability and respiratory efficiency due to resistance of the soft tissue and maxillary sutures around the nose. So it is essential that the professional should consider the possible consequences for the soft tissue as a result of this treatment.^9^, ^23^, ^36^

The vast majority of studies existing in the literature have approached skeletal and soft tissue alterations by means of two-dimensional examinations^5^, ^6^, ^11^, ^14^, ^15^, ^16^, ^17^, ^18^, ^20^, ^21^, ^29^, ^30^, ^32^, ^33^ however technological advances in digital imaging have made it possible to perform these evaluations in 3D. Computed tomography is a reliable method for representing and measuring the thickness of facial soft and skeletal tissue^37^, ^38^ providing highly precise and accurate data, producing full-size anatomical images (1:1). The majority of studies that use three-dimensional examinations,^7^, ^19^, ^22^, ^23^, ^24^, ^25^, ^26^, ^27^, ^28^, ^30^, ^31^, ^35^, ^36^, ^39^, ^40^ however, have not explored the study of the nasal cavity in three dimensions, instead placing greater emphasis on alterations in width.^19^, ^20^, ^21^, ^22^, ^23^, ^24^, ^25^, ^26^, ^28^, ^30^, ^39^

Due to the conflicting literature and the lack of information about three-dimensional skeletal alterations, and mainly through the absence of a more comprehensive literature in terms of soft tissue nasal alterations, the objective of the present study is to evaluate, using a simple but reliable and reproducible methodology the effects of RME on the skeletal and soft tissue structures of the nose, in three dimensions, in mouth-breathing patients, with the assistance of computed tomography.

## Methods

The sample comprised 55 mouth-breathing individuals suffering from maxillary constriction, referred for RME treatment. The patients were divided into the experimental group (EG) (39 patients–23 male and 16 female with an average age of 9.7 years, standard deviation of 2.28, ranging from 6.5 to 14.7 years) and a Control Group (CG) (16 patients – 9 male and 7 female with an average age of 8.8 years, standard deviation of 2.17, ranging from 5 to 13.7 years). All patients were evaluated by a multi-disciplinary team and the diagnoses were made through a standardized questionnaire, and ENT and orthodontic evaluation. Syndromic patients or patients with craniofacial abnormalities such as Pierre Robin and Treacher Collins, among others, and patients with dental or periodontal changes were excluded from the study. This study was approved by the Committee for Ethics in Institutional Research.

The patients of the EG were treated by using the Hyrax maxillary expander with six-quarter initial activations and two-quarter daily activations until the necessary quantity of expansion was obtained for each patient, in other words, until the superior buccal bone beseem compatible with the inferior WALA edge. Once the activations were complete, the appliances were kept in place for a period of 3 months, as a retainer, to enable new bone to forming the region of the mid-palatal suture. CT examinations were conducted at two different points in time: (T1) pre-RME and (T2) three months after the retainer is fitted. The patients in the CG were subjected to the same CT examinations (T1 and T2) on similar periods to those in the EG (three months between them). All the patients in the CG were duly treated using the same standard procedure employed in the EG, once the study was over. All the CT scans were performed in the same location and with the same equipment, namely: Multislice (Philips Brilliance CT 64 channel scanner). For these examinations, a FOV of 20 cm and Voxel of 0.25 mm were used. All patients were under medical treatment, all TC scans were part of the diagnosis process and by medical prescription, respecting the ALARA principle according to the skull size of each patient.

**Figure 1:**
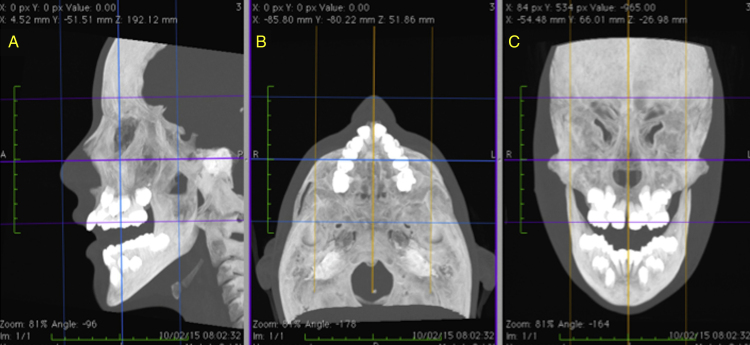
Final repositioning of the head with the sagittal (A), axial (B) and coronal (C) slices duly oriented in relation to the Frankfurt plane and the midsagittal plane.

The skeletal and soft tissue structures of the nose were duly manipulated and measured in three dimensions based on anatomical points defined in the global literature^36^, ^40^, ^41^, ^42^ (Table 1, Table 2), with the aid of OsiriX MD software (FDA approved, version 1.4.2; Pixmeo, Geneva, Switzerland), which, by means of specific tools, permitted the acquisition of multiplanar slices (sagittal, axial and coronal) of CT images, through which the data were collected. In order to assure the correct localization of the anatomical points,^41^, ^42^ good reproducibility and reliable measurements, prior to measuring, the repositioning of the head was carried out for all the CT mages, following the methodology advocated by Cevidanes et al.^43^ (Fig. 1A–C).

**Table 1:** Points located in the skeletal tissue.

Nasion (N)	Intersection of the frontal bone and two nasal bones.
Anterior Nasal Spine (ANS)	Most anterior point of the maxilla's nasal spine.
Posterior Nasal Spine (PNS)	Most posterior point of the maxilla's nasal spine.

**Table 2:** Points located in the soft tissue.

Nasion (N′)	Point of the midline of the soft tissue that lies directly over the hard tissue, in the direction of the skeletal Nasion (N).
Pronasal (Prn)	Most prominent point of the nose located on the median.
Alar (Al)	The most lateral point of the contour of each nostril.
Alar Curvature (Ac)	Point located at the soft tissue insertion of each alar base.
Subnasal (Sn)	Mid-point between the meeting of the lower edge of the nasal septum and upper lip, located on the median.

**Figure 2:**
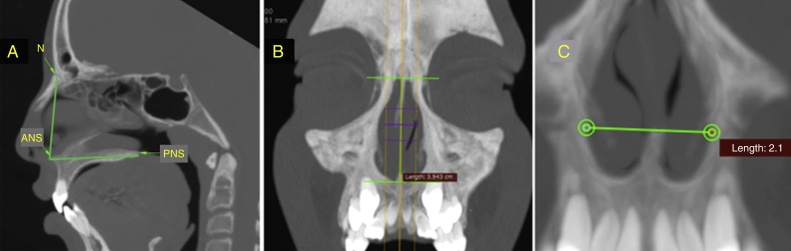
(A) Measurement of the nasal height (N-ANS) and total length (ANS-PNS) in the sagittal slice. (B) Measurement of the height of the pyriform aperture in the coronal slice. (C) Measurement of the width of the pyriform aperture in the coronal slice.

The skeletal structures were measured in the sagittal and coronal images of the multiplanar slices. In the sagittal image (Fig. 2A), the nasal height is measured via the measurement of linear distance (mm) between points N and ANS and the total length of the nose via the distance, in millimeters, between the points ANS and PNS. As for the coronal image, the height of the pyriform aperture is measured, obtained via the linear distance (mm) between the upper and lower tangents of this structure above the midsagittal plane (Fig. 2B) and the nasal width through the distance (mm) between the outermost points of the lateral walls of the lower third of the pyriform aperture (Fig. 2C).

**Figure 3:**
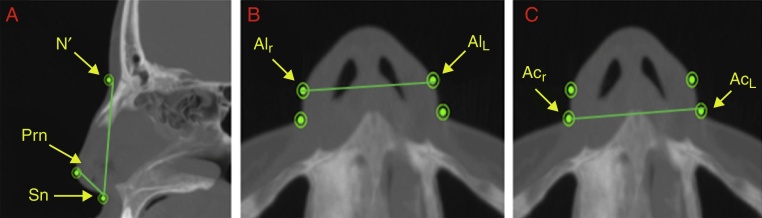
(A) Measurement of the height of the nasal soft tissue (N′–Sn) and length of the nasal soft tissue (Prn–Sn) in the sagittal slice. (B) Measurement of the alar width (Al_r_–Al_l_) in the axial slice. (C) Measurement of the width of the soft tissue insertion (Ac_r_–Ac_l_) in the axial slice.

**Figure 4:**
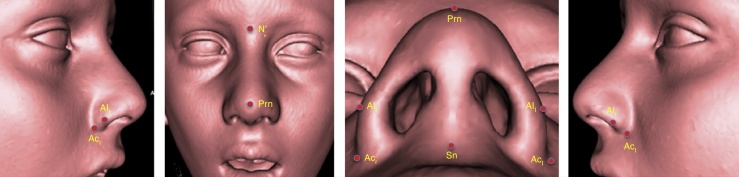
Points in 3D soft tissue reconstruction.

The soft tissue measurements were measured in the sagittal and axial images of the multiplanar slices. The height was obtained in the sagittal image via the linear distance (mm) between the points N′ and Sn (Fig. 3A) and the length, also in the sagittal image, via the linear distance (mm) between the points Prn and Sn (Fig. 3A). The width of the nasal soft tissue was obtained from the axial image via the linear distance (mm) between the points Al_r_ and Al_L_ (alar width) and between the points Ac_r_ and Ac_L_ (width of the soft tissue insertion) (Fig. 3B and C). Fig. 4 shows the same points referred to 3D reconstruction.

### Statistical analysis

The statistical analysis and data treatment was performed using the program Statistical Package for the Social Sciences (SPSS), version 22 for Windows. The measurements are shown in millimeters (mm) and described via the mean (M) and standard deviation (SD), the results being displayed in the format *M* ± DP.

In order to verify the adequacy of the sample for the study, Student's *t*-tests were performed for paired samples. The calculations were carried out using the program G*Power and showed a sample (*n* = 55) with power of 80% at a 5% level of significance, demonstrating that the sample is sufficient to assure the credibility of the results obtained.

The significance values (*p*) of the Shapiro–Wilk test for the analysis of normality of data were greater than or equal to 0.05 for all variables. These results with a 5% level of significance permit us to assume that the data have a normal distribution, guaranteeing the prerequisites for the performance of parametric tests for the statistical analysis and treatment of the results obtained in this study.

In order to check for the possibility of potential interference from gender and age, in the results of the study, the Fisher's exact test was used for the gender variable (58.2% male and 41.8% female) and the independent Student's *t*-test for the age variable (in the global sample the ages range from 5.92 to 14.17 years, with *M* = 9.45 and SD = 2.26). The test results demonstrate there were no significant differences either in terms of gender (*p* = 1.000) or age (*p* = 0.191). Based on these results, it may be stated that, despite there being more males than females and a large age variation between the sample elements, there was no interference from either of these two factors in the outcome of the study.

In order to analyze method error, intra-examiner reliability was assessed using the Student's *t*-test for paired samples and the Intraclass Correlation Coefficient (ICC). All the pre- and post-RME CT examinations were measured again 30 days after the initial measurements, assuming a level of significance of 5% for the decision with regard to the outcome of the statistical tests. The results showed that there are no significant differences (*p* > 0.05) between the means of the first and second measurements in any of the soft tissue or skeletal variables, nor in the pre- and post-RME tomography. The ICC values were above 0.05 for all variables, indicating an excellent consistency between the results of the first and second measurements, assuring excellent intra-examiner reliability.

For the analysis of the inter-examiner method error a total of 27 patients were selected at random (total of 54 CTs) out of the 39 patients in the EG sample who were measured by a second examiner. The Student's *t*-test for paired samples detected no statistically significant differences (*p* > 0.05) between the means of the first examiner and the means of the second examiner for any of the variables nor in the pre- and post-RME tomography. The ICC values were in excess of 0.95 for all variables, indicating excellent consistency between the results of the first and second measurements, assuring excellent inter-examiner reliability.

For the analysis of the comparison between T1 and T2 values in both groups, for the skeletal and soft tissue variables, and for the comparison between the groups, the Student's *t*-test for paired samples was used.

## Results

All the CT images were measured twice by the same examiner at an interval of 30 days. The CT's were numbered so that the examiner would not know if he was measuring images from the pre- or post-RME images. After the CT measurement, the CT's were arranged into (T1) pre- and (T2) post-RME. The same procedure was used for the CT scans of the CG.

The evaluation of the results on the skeletal and soft tissue structures in both groups was conducted using the values of the T1 and T2 measurements and the results for the skeletal alterations are displayed in Table 3 and the results for the soft tissue variables are shown in Table 4.

**Table 3:** Comparison between pre-RME and post-RME values, and between the Experimental Group (EG) and Control Group (CG), of the skeletal variables (values displayed in mm).

Variable	Group	Pre-RME *M* ± DP (Min–Max)	Post-RME *M* ± DP (Min–Max)	Pre-RME post-RME alteration (average)		*p*^a^ (pre-RME–post-RME)
				mm	%	
Nasal height	EG	44.81 ± 4.01 (38.37–57.34)	46.32 ± 3.94 (40.23–57.14)	+1.51	+3.37%	<0.001
	CG	44.93 ± 4.35 (38.01–52.13)	45.05 ± 4.21 (38.41–52.91)	+0.12	+0.27%	0.537
*p*^b^ (between groups)		0.920	0.003			

Height of pyriform aperture	EG	31.52 ± 3.42 (22.66–37.15)	32.78 ± 3.40 (24.72–39.33)	+1.26	+4.00%	< 0.001
	CG	31.24 ± 3.51 (25.05–37.26)	31.14 ± 3.24 (25.40–37.98)	−0.10	−0.32%	0.776
*p*^b^ (between groups)		0.790	0.003			

Width of pyriform aperture	EG	21.64 ± 1.73 (17.83–25.34)	23.62 ± 2.45 (18.19–31.39)	+1.98	+9.15%	< 0.001
	CG	21.28 ± 1.98 (17.52–24.55)	21.28 ± 1.88 (17.83–24.41)	0.00	0.00%	0.985
*p*^b^ (between groups)		0.503	0.001			

Total length of the nose	EG	47.25 ± 3.35 (37.76–54.61)	47.78 ± 3.53 (37.18–54.87)	+0.53	+1.12%	0.002
	CG	47.50 ± 3.09 (41.22–53.16)	47.71 ± 2.83 (42.08–52.75)	+0.21	+0.44%	0.444
*p*^b^ (between groups)		0.796	0.938			

a *p*, significance value of Student's *t*-test for paired samples (difference between pre and post).

b *p*, significance value of Student's *t*-test for independent samples (difference between the EG and the CG).

**Table 4:** Comparison between pre-RME and post-RME values and between the Experimental Group (EG) and Control Group (CG), of the soft tissue variables (values displayed in mm).

Variable	Group	Pre-RME *M* ± DP (Min–Max)	Post-RME *M* ± DP (Min–Max)	Pre-RME Post-RME alteration (average)		*p*^a^ (pre-RME–post-RME)
				mm	%	
Width of the soft tissue insertion	EG	32.59 ± 2.84 (28.73–39.95)	34.02 ± 2.85 (29.58–41.99)	+1.43	+4.39%	< 0.001
	CG	32.71 ± 1.72 (30.09–35.44)	32.78 ± 1.89 (29.88–36.19)	+0.07	+0.21%	0.758
*p*^b^ (between groups)		0.876	0.002			

Alar width	EG	32.68 ± 3.27 (27.13–40.59)	33.81 ± 3.32 (27.43–42.75)	+1.13	+3.46%	< 0.001
	CG	32.69 ± 1.74 (30.16–35.81)	32.29 ± 2.08 (29.13–36.31)	−0.40	−1.22%	0.085
*p*^b^ (between groups)		0.990	0.003			

Height of the nasal soft tissue	EG	47.95 ± 4.07 (41.27–58.77)	49.74 ± 4.39 (41.70–59.14)	+1.79	+3.73%	< 0.001
	CG	47.49 ± 4.69 (40.16–55.89)	47.69 ± 4.67 (40.41–55.86)	+0.20	+0.42%	0.101
*p*^b^ (between groups)		0.714	0.003			

Length of the nasal soft tissue	EG	15.89 ± 1.62 (12.17–18.84)	16.57 ± 1.67 (12.38–20.68)	+0.68	+4.28%	< 0.001
	CG	15.41 ± 1.38 (13.30–17.74)	15.66 ± 1.57 (12.05–18.29)	+0.25	+1.62%	0.161
*p*^b^ (between groups)		0.301	0.068			

a *p*, significance value of Student's *t*-test for paired samples (difference between pre and post).

b *p*, significance value of Student's *t*-test for independent samples (difference between the EG and the CG).

For the skeletal variables, in the EG, a significant increase (*p* < 0.05) was observed in all the structures. The largest increase was found with the width of the pyriform aperture (+9.15%), followed by the height of the pyriform aperture (+4.00%), nasal height (+3.37%) and the total length of the nose (+1.12%). Among the patients of the CG, no significant changes were observed. When comparing the EG with the CG, the most significant difference was found in the width of the pyriform aperture, post-RME (*p* = 0.001). Changes in total nose length were not significant in the comparison between groups.

With regard to the soft tissue variables, it was also found, in the EG, a significant increase (*p* < 0.05) in all the analyzed structures between the points in time T1 and T2. In percentage terms, the increase was +4.39% in the width of the soft tissue insertion, +3.46% in the alar width, +3.73% in the height of the nasal soft tissue and +4.28% in the length of the nasal soft tissue. Among the patients of the CG, no significant changes were observed. When comparing the EG with the CG, the greatest difference was found in the width of the soft tissue insertion (*p* = 0.002) and the length of the nasal soft tissue did not show significant changes.

The analysis of the ratios between the skeletal and soft tissue measurements, in the EG, showed that the alterations occur in a proportion close to 1:1. The mean of the alterations of the 4 soft tissue variables was divided by the mean of the alterations in the 4 skeletal measurements. The outcome allows us to state that, for each millimeter increase in the skeletal variables, the soft tissue variables increase 0.95 mm. In percentage terms, for each percentage unit increase in the skeletal measurement, the soft tissue measurements increase 0.9%.

## Discussion

Since RME was introduced in 1860, becoming established in the period between the end of the 1860s^8^ and beginning of the 1870s,^9^ orthodontists have extensively evaluated this procedure with regard to dentoskeletal alterations and the repercussions for the nasal cavity. The close relationship between the maxilla and the nasal cavity^5^, ^6^ may have an influence on the physiology^1^, ^2^ and format of the nose,^10^ increasing the chances of individuals with maxillary hypoplasia acquiring a clinical mouth-breathing condition^10^ which may have serious consequences for the growth and development of the face and occlusion,^2^, ^3^, ^4^, ^7^ besides problems of a medical nature, and on the development of the body.^3^, ^12^, ^13^ Accordingly, various researchers have sought to shed light on the possible benefits of RME for the nasal cavity and on the increase in nasal breathing capacity.^1^, ^2^, ^7^, ^11^

In recent years, the soft tissue of the nose has been cited by some researchers as being a structure of extreme importance for maintaining the stability of the results obtained with RME,^23^, ^33^ in addition to the esthetic impact that may ensue after the procedure.^36^ Even so, studies dealing with these structures are scarce, and the main focus of almost all the works cited in the literature is on skeletal alteration.

The proposal in our study was, using CT, to evaluate the repercussions of RME on the skeletal and soft tissue structures of the nose in mouth-breathing patients with maxillary hypoplasia. CT has been shown to be an excellent diagnostic resource providing conditions for locating anatomic points with a high degree of reliability, as well as providing measurements of soft and skeletal tissue with extreme precision.^38^

Patients treated with RME present with transverse deficiencies of the maxilla and, therefore, additional transverse alterations can be expected. The results showed, however, that after RME, changes occurred in the 3 dimensions of the nose and not just the width, which has been the main focus of the majority of the studies reviewed.

With regard to skeletal alterations, our studies found larger alterations in the width of the pyriform aperture with a significant average increase (*p* < 0.001) of 1.98 mm (+9.15%). Similar results were obtained by Hershey et al. (+2.12 mm),^15^ Sila Filho et al. (+2.07 mm),^16^ Chung and Font (+1.75 mm),^5^ Garret et al. (+1.89 mm),^18^ Iwasaki et al. (+2.09 mm),^25^ Bargazani et al. (1.4–2.7 mm),^27^ Hakan and Palomo (+1.5 mm),^30^ and Leri and Basciftci (+1.97 mm).^32^ Larger increases in nasal width, also significant, were observed by Wertz (+4 mm),^14^ Basciftci and Karaman (+3.47 mm),^18^ Christie et al. (+2.73 mm),^21^ Görgülü et al. (+5.28 mm),^22^ Smith et al. (+3.15 mm)^23^ and Toku et al. (+2.54 mm).^31^ The studies conducted by Cross and McDonald (+1.06 mm),^17^ Ballanti et al. (+1.22 mm),^20^ Ribeiro et al. (+1.28 mm),^26^ Kanomi et al. (+1.36 mm)^28^ and Çörekçi and Göyenç (+0.81 mm)^29^ also showed increases in nasal width, however only the study by Cross and McDonald^17^ exhibited any statistical significance. The largest differences noted occurred in studies that adopted methodologies which used two-dimensional diagnosis.^14^, ^29^ Of the three-dimensional methodologies employed, only the study by Görgülü et al.^22^ found larger deviations than the means of the other studies.

The analysis of possible alterations in the height of the nasal cavity, showed that in sagittal form (multiplanar sagittal image) nasal height increased significantly (*p* < 0.001) by 1.51 mm (+3.37%) and in frontal form (multiplanar coronal image) the height of the pyriform aperture showed significant average increases (*p* < 0.001) of 1.26 mm (4%). The only works that evaluated skeletal alterations in the height of the nasal cavity, post-RME, were those of Cross and McDonald (+1.26 mm)^17^ and Çörekçi and Göyenç (+1.35 mm).^29^ Both used two-dimensional methodologies in frontal form.

We also evaluated the total length of the nasal skeleton, noting a significant increase (*p* < 0.002) of 0.53 mm (+1.12%). None of the evaluated works researched potential alterations in the skeletal length of the nasal cavity.

All the studies surveyed in the literature performed short-term evaluations (between 3 and 6 months on average) and, as with the results of our study, a large part of them found there to be no influence on growth by virtue of the short space of time between pre- and post-RME evaluations,^7^, ^10^, ^11^, ^14^, ^20^, ^30^, ^32^ contradicting the findings of Silva Filho et al.,^16^ Cross and McDonald,^17^ Kanomi et al.^28^ who determined that, even in the short term, the growth factor could affect the structures being measured.

In making a short-term study, the goal was to analyze the real nasal alterations from specifically the actions of the REM at the evaluated structures, avoiding that the growth factor would interfere in the results if evaluated in long term.

Some authors checked for skeletal modifications by means of ENT examinations,^1^, ^2^, ^7^, ^11^ with the aim of evaluating the real benefits of RME on the nasal physiology and found that, in the main, the alterations in the width of the nasal cavity contribute significantly to increased flow and nasal airway volume in patients with maxillary hypoplasia who had been subjected to RME treatment.

In the evaluation of the soft tissue structures of the nose, in the EG, the largest increases found in our study occurred in the width of the soft tissue insertion with an average of +1.43 mm (+4.39%) (*p* < 0.001). The alar width increased on a slightly lower scale at 1.13 mm (+3.46%), though with the same statistical significance (*p* < 0.001). Several of the albeit few studies found in the literature that deal with alterations in the width of the soft tissue of the nose, post-RME, have found values similar to those in the present study, for example Berger et al. (+2 mm),^33^ Johnson et al. (+1.5 mm)^37^ and Ylmaz et al. (+1.19 mm),^40^ and others exhibited higher values like the studies of Kulbersh et al. (+3.2 mm)^39^ and Magnusson et al. (+2.88 mm)^36^ or much lower values, as is the case with Kim et al. (+0.96 mm),^35^ though all demonstrated statistically significance.

By evaluating the length of the nasal soft tissue, we found a significant average increase (*p* < 0.001) of 0.68 mm (+4.28%) in the EG, post-RME. The studies by Karaman et al. (+2.53 mm)^34^ and Magnusson et al. (+3.09 mm)^36^ exhibited greater increases while Ylmaz et al. detected lower increases (+0.31 mm),^40^ however all were significant, contradicting the results of Kiliç et al.^6^ whose increase of 0.23 mm in the soft tissue length was deemed insignificant (*p* > 0.05).

In a study of the potential alterations in the height of the nasal soft tissue, post-RME, we found in the EG a significant average increase (*p* < 0.001) of 1.79 mm (+3.73%). The only study that dealt with the height of the nasal soft tissue was that of Magnusson et al.^36^ with an insignificant average (*p* > 0.05) of +0.18 mm, but these authors analyzed the alterations occurred after surgery disjunction and not a RME as in our study.

The only study on soft tissue alterations which, like the present study, studied modifications to the nose in three dimensions, was that of Magnussen et al.^36^ as previously dimensioned, a work based on surgical expansion which makes our study the first to take this approach with patients who have been exclusively treated with orthopedic RME.

As with those studies that evaluated skeletal alterations, all the works on soft tissue were performed over a short period of time,^6^, ^33^, ^37^, ^40^ showing all alterations occurred exclusively by the effect of the RME without the influence of the growth factor due to the short interval between T1 and T2.

According to Berger et al.^33^ and Kulbersh et al.,^39^ the soft tissue tends to accompany RME skeletal alterations in a 1:1 proportion (100%). These values closely approximate the results of our study which found that, for every 1 mm of skeletal increase, 0.5 mm of soft tissue alteration takes place (0.90%), contradicting the studies of Karaman et al.,^34^ which state that there is only 18% of soft tissue alteration during skeletal modifications.

An essential factor in performing a correct analysis is the reliability of the measurements, i.e., the precision of the selected points^41^, ^42^, ^43^ and the analysis of the method error in the measurements. Our study demonstrated a high reliability with ICC values in excess of 0.95, both for the intra-examiner and the inter-examiner evaluations. For the most part, the studies quoted in the literature did not display method error and several did not present a sampling calculation, to validate the size of the sample, which may lead to bias in the results and interpretation.

The results obtained in the EG made it clear that post-RME nasal changes occur in the three dimensions, both in soft tissues and in skeletal tissues, but the comparison between the EG with the CG showed that the changes in length are those that have the less significant impact among the evaluated structures. In addition, the statistical results obtained in the CG, when comparing T1 and T2 times, showed that there was no interference of the growth factor between the evaluation periods.

We should remember again that all patients that took part of this research were under medical treatment in hospital level and all CT exams, an the intervals between them were undertaken by exclusive medical needs, respecting the ALARA principle to each patient. Furthermore, it is also necessary to clarify that no medical conduct was performed prior to RME, i.e., the medical conducts were determined only after RME, therefore, none of these patients in the sample were underwent to any medical/surgical procedure that could change the characteristics of the nasal soft tissues within this time interval.

It is also important to clarify that, after the end of the study, the patients in the CG were properly treated with the same procedures of the EG, without any prejudice to them, due to the small time of three months between T1 and T2 times.

Our study utilized an already existing database with the pertinent authorizations and sought to follow all the steps required to guarantee maximum credibility with the results obtained, ranging from the use of a simple methodology that is reliable and easily reproducible to the performance of all the necessary statistical studies to diminish the risk of bias, thereby increasing the reliability of the study, duly approved by the research ethics committee and by the Clinical Trials (ID: CRB-ORTO-3). This kind of study may permit in the future, through the evaluation of soft tissue, adequate planning of esthetic procedures at the tip and base of the nose and also the performance of objective measurements in early or late surgical results.

## Conclusion

1. In the short term, RME caused significant skeletal and soft tissue alterations in all the variables studied when analyzing pre- and post-RME measurements.
2. The soft tissues of the nose accompany the skeletal alterations in a proportion close to 1:1, and for every millimeter of skeletal increase there is a soft tissue increase of 0.95 mm (0.90%).
3. This kind of study may permit in the future, through the evaluation of soft tissue, adequate planning of esthetic procedures at the tip and base of the nose and also the performance of objective measurements in early or late surgical results.

## Conflicts of interest

The authors declare no conflicts of interest.
